# Supplementary material for: Comparative analysis of transferrin and IgG N-glycosylation in two human populations
Source: Commun Biol. 2023 Mar 23;6:312. doi: 10.1038/s42003-023-04685-6 (PMC10036557; doi:10.1038/s42003-023-04685-6)
Supplement: Supplementary file 2 — Supplementary Information [file 42003_2023_4685_MOESM2_ESM.pdf]

COMPARATIVE ANALYSIS OF TRANSFERRIN AND IgG N-GLYCOSYLATION IN TWO HUMAN POPULATIONS

Irena Trbojević-Akmačić[1,#], Frano Vučković[1,#], Tea Pribić[1], Marija Vilaj[1], Urh Černigoj[2], Jana Vidič[2], Jelena Šimunović[1], Agnieszka Kępka[1,3], Ivana Kolčić[4,5], Lucija Klarić[6], Mislav Novokmet[1], Maja Pučić-Baković[1], Erdmann Rapp[7,8], Aleš Štrancar[2], Ozren Polašek[4,5], James F. Wilson[6,9], Gordan Lauc[1,10]

[1]Genos Glycoscience Research Laboratory, Zagreb, Croatia

[2]BIA Separations d.o.o., a Sartorius company, Ajdovščina, Slovenia

[3]University of Warsaw, Faculty of Biology, Institute of Zoology, Department of Immunology, Warsaw, Poland

[4]Department of Public Health, University of Split School of Medicine, Split, Croatia

[5]Algebra University College, Zagreb, Croatia

[6]MRC Human Genetics Unit, Institute for Genetics and Cancer, University of Edinburgh, Edinburgh, United Kingdom

[7]Max Planck Institute for Dynamics of Complex Technical Systems, Magdeburg, Germany

[8]glyXera GmbH, Magdeburg, Germany

[9]Centre for Global Health Research, Usher Institute, University of Edinburgh, Edinburgh, United Kingdom

[10]University of Zagreb, Faculty of Pharmacy and Biochemistry, Zagreb, Croatia

# = These authors contributed equally

Supplementary Figures

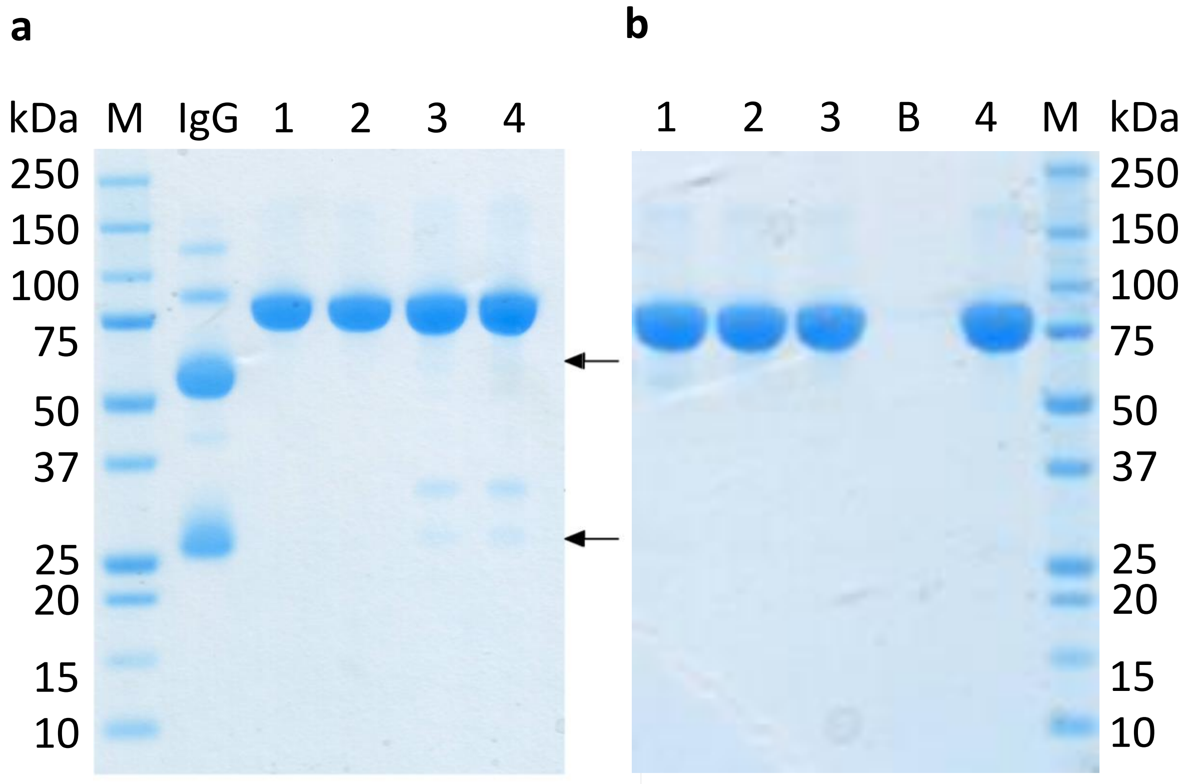

Supplementary Figure 1. Transferrin (Tf) purity analyzed by SDS-PAGE under reducing conditions after high-throughput isolation from human plasma. a, Elution fractions (5 × concentrated) after Tf (around 80 kDa) isolation from immunoglobulin G (IgG) depleted plasma (lanes 1 and 2) and plasma without IgG depletion (lanes 3 and 4). Lane IgG - IgG sample with two prominent bands corresponding to the heavy chain and light chain, while the upper bands represent incompletely reduced IgG. Arrows represent bands from contaminants in the case when IgG was not removed from blood plasma before Tf isolation. **b,** Elution fractions (7.5 × concentrated) after Tf isolation from IgG depleted plasma (lanes 1-4). B - blank sample (10 × concentrated). M - Precision Plus Protein Standards All Blue molecular mass standard (Bio-Rad). Protein bands were visualized by GelCode Blue staining reagent.

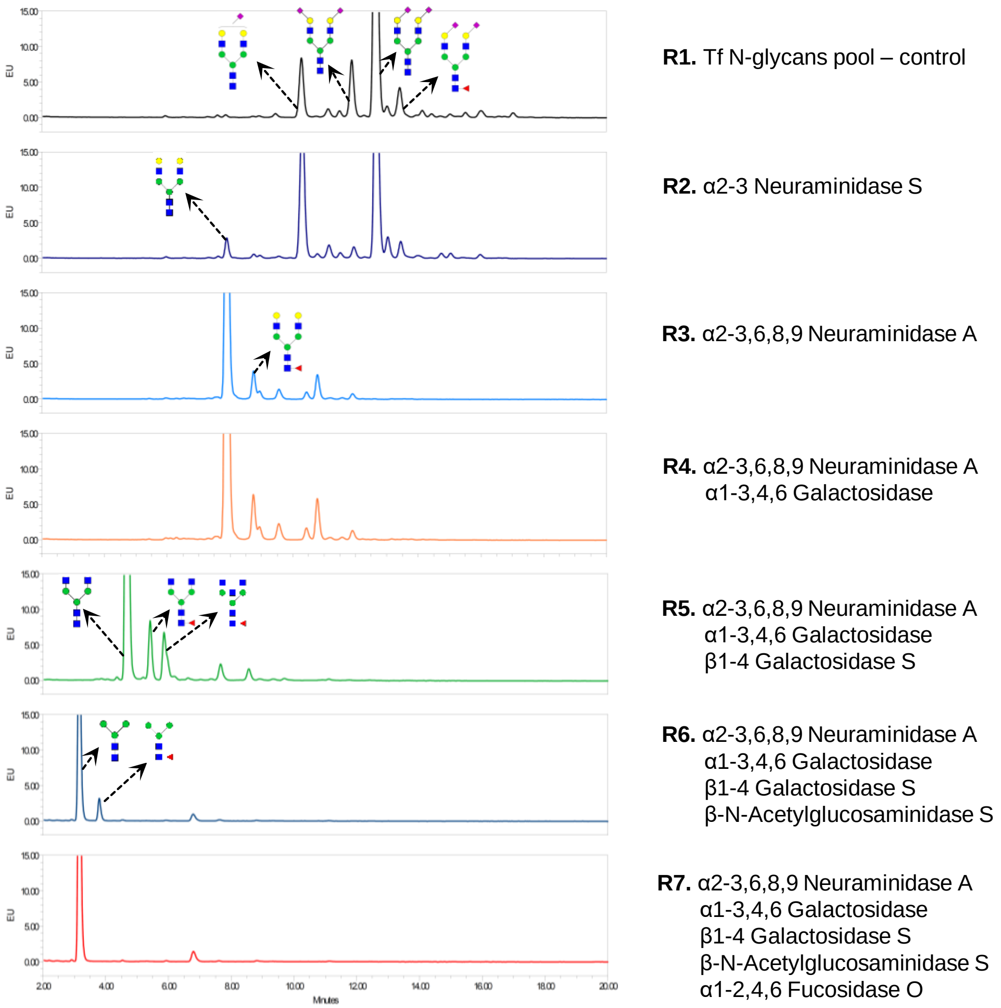

Supplementary Figure 2. **Exoglycosidase sequencing of transferrin (Tf) N-glycome in seven reactions containing different mixtures of exoglycosidases (R1-R7).** Details of reactions are provided in **Supplementary Table 3**. EU – emission units.

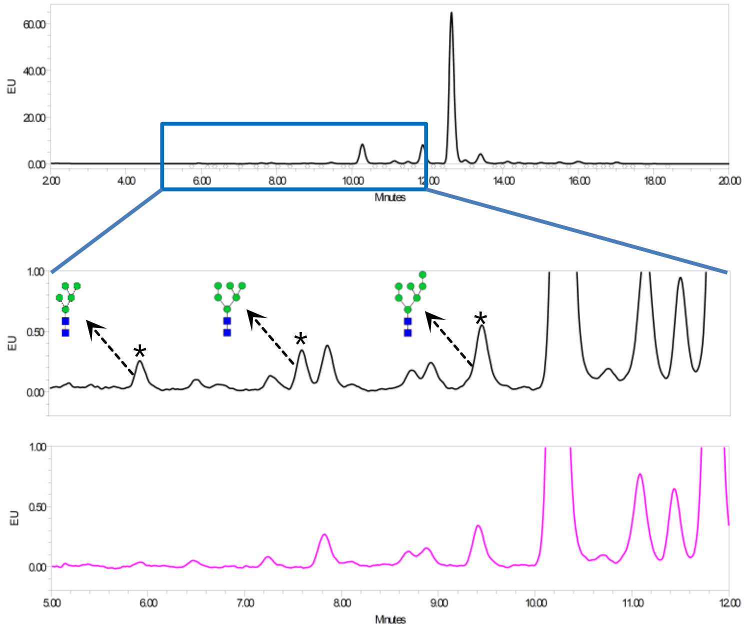

R1. Tf N-glycans pool – control

R1. Tf N-glycans pool – control (zoomed in view)

R8. α1-2,3,6 Mannosidase (zoomed in view)

Supplementary Figure 3. **Exoglycosidase sequencing of transferrin (Tf) N-glycome in two reactions containing different mixtures of exoglycosidases (R1 and R8).** Details of reactions are provided in **Supplementary Table 3**. EU – emission units.

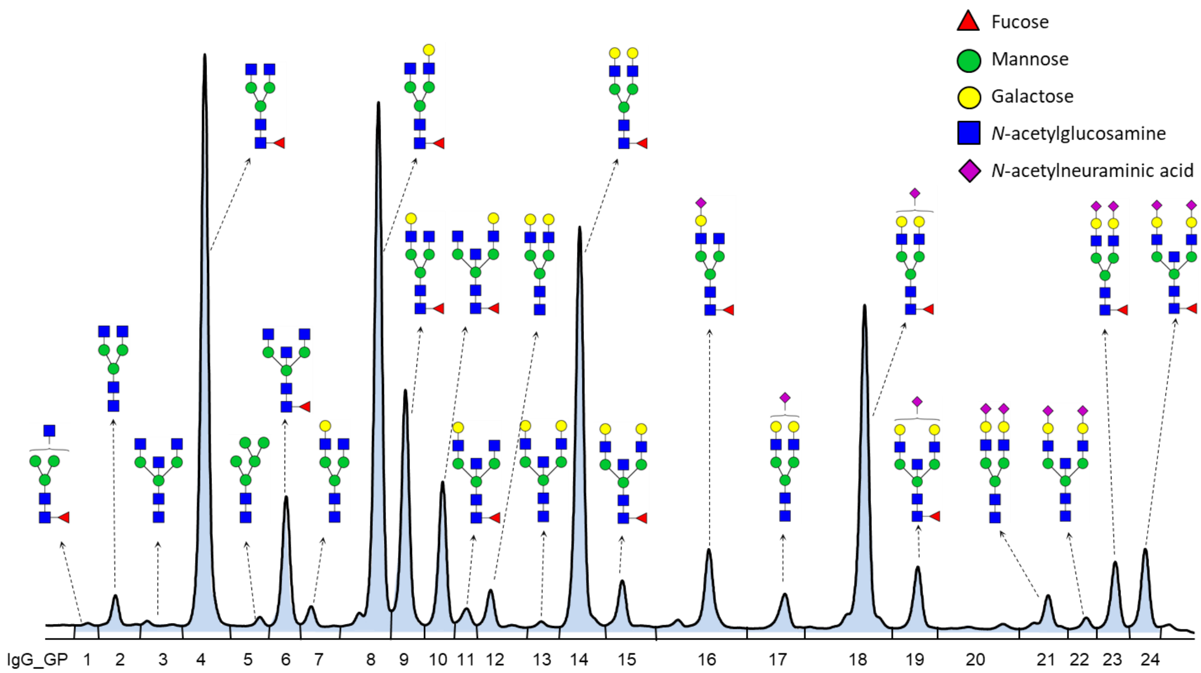

Supplementary Figure 4. N-glycosylation profile of human plasma immunoglobulin G (IgG) obtained by ultra-high-performance liquid chromatography based on hydrophilic interactions with fluorescent detection (HILIC-UHPLC-FLD). The most abundant glycan structure in each IgG glycan peak (IgG_GP) is shown [1]. Structural schemes are given according to Consortium for Functional Glycomics (CFG) guidelines.

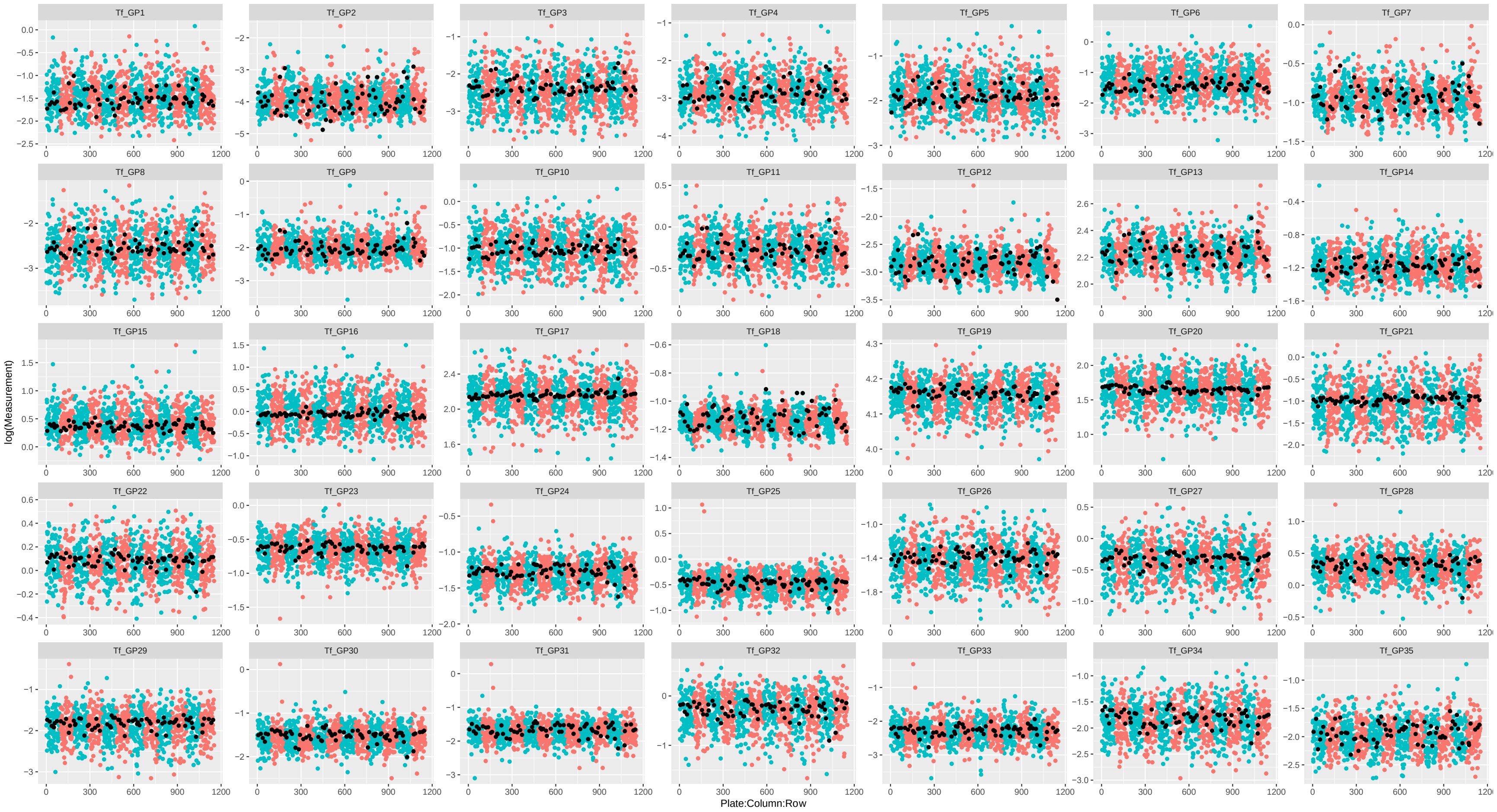

Supplementary Figure 5. Transferrin (Tf) N-glycome measured by HILIC-UHPLC-FLD in Korcula cohort. Normalized, log-transformed, and batch-corrected data are shown. Each panel represents one of the Tf N-glycan peaks (Tf_GP1-Tf_GP35). Black dots represent values obtained for internal standard samples. Red and turquoise dots represent biological samples analyzed in different batches (turquoise – odd-numbered batches; red – even-numbered batches).

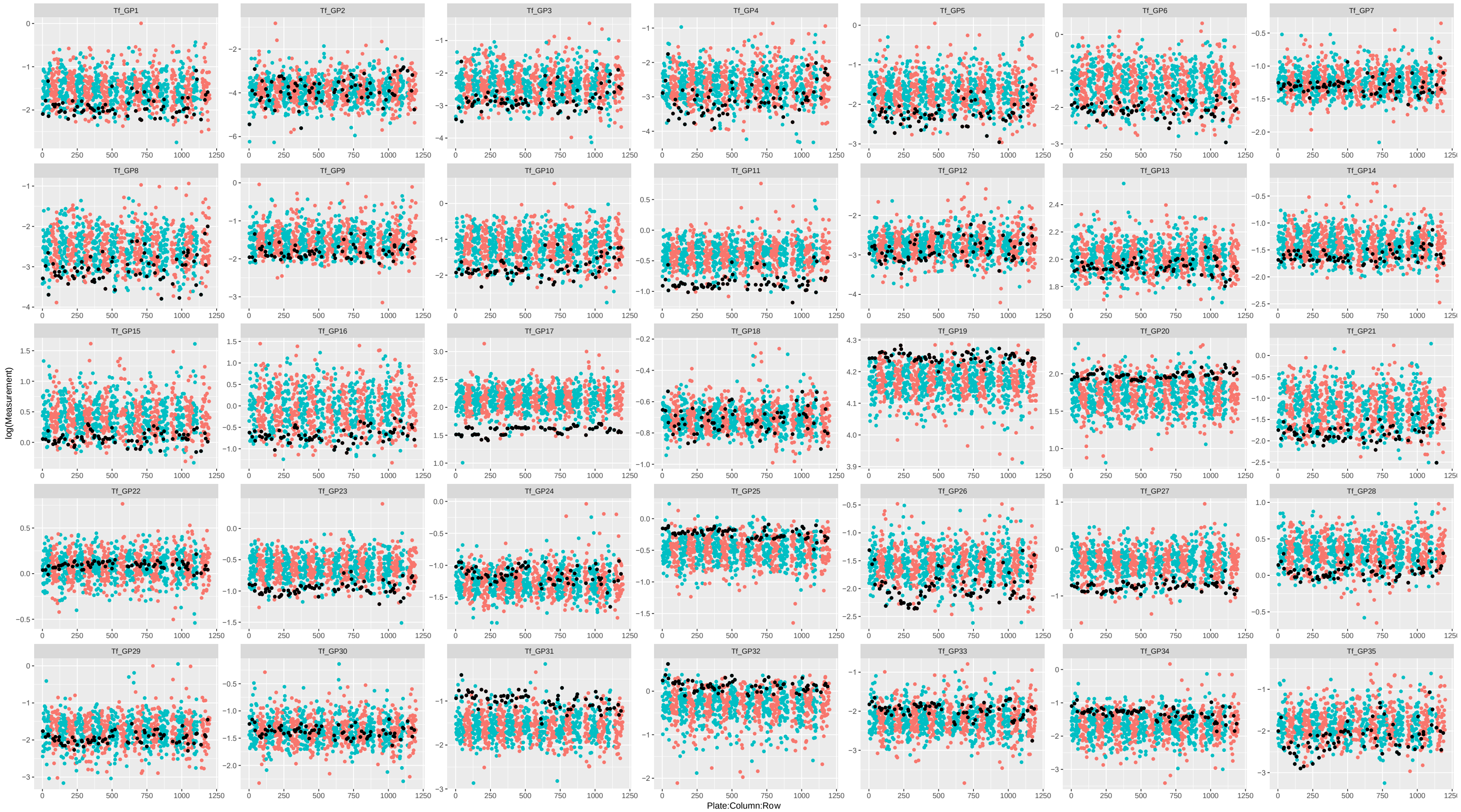

Supplementary Figure 6. Transferrin (Tf) N-glycome measured by HILIC-UHPLC-FLD in VIKING cohort. Normalized, log-transformed, and batch-corrected data are shown. Each panel represents one of the Tf N-glycan peaks (TfGP1-TfGP35). Black dots represent values obtained for internal standard samples. Red and turquoise dots represent biological samples analyzed in different batches (turquoise – odd-numbered batches; red – even-numbered batches).

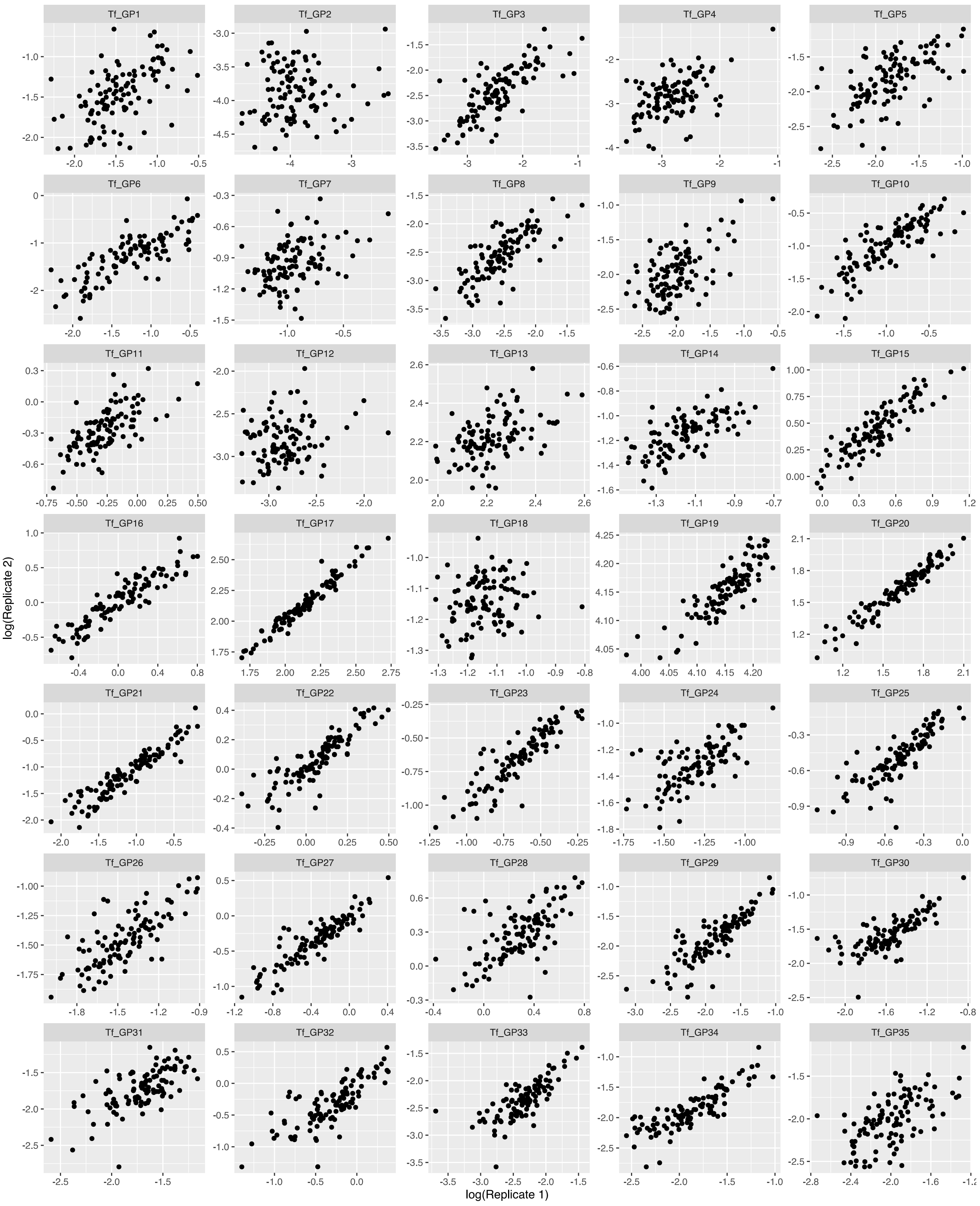

Supplementary Figure 7. Correlation of transferrin (Tf) N-glycome measured by HILIC-UHPLC-FLD in duplicate samples of Korcula cohort. Replicate 1 is plotted on the x-axis and Replicate 2 on the y-axis. Normalized, log-transformed, and batch-corrected data are shown. Each panel represents one of the Tf N-glycan peaks (Tf_GP1-Tf_GP35).

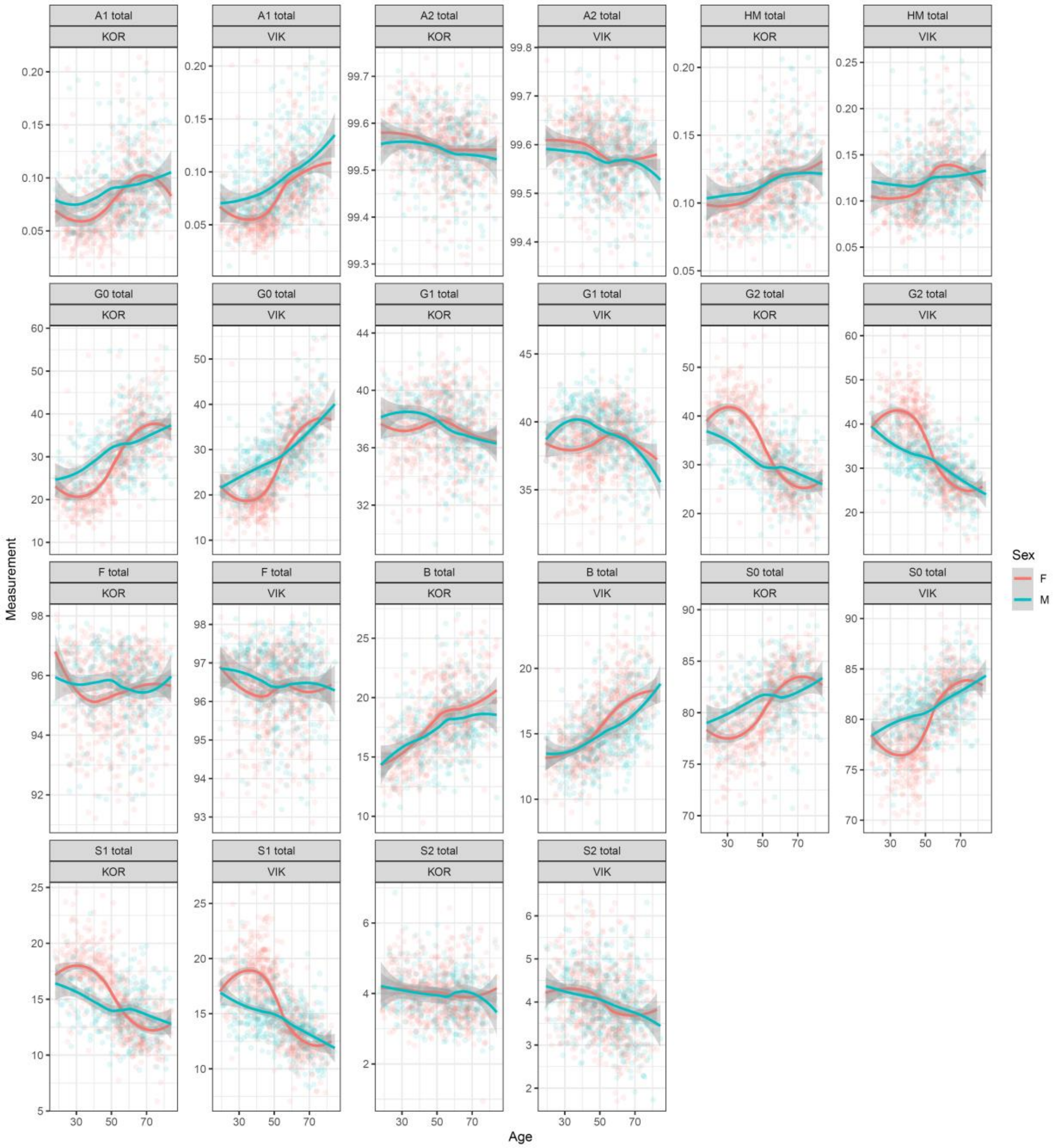

Supplementary Figure 8. N-glycosylation profile of human plasma immunoglobulin G (IgG) is associated with age and sex. Derived N-glycosylation traits are shown for each cohort (KOR – Korcula, VIK – VIKING) and calculations are given in **Supplementary Table 5**. Turquoise and red curves are fitted local regression models describing a sex-specific relationship between age and derived traits. The grey-shaded region is a pointwise 95% confidence interval on the fitted values (there is 95% confidence that the true regression curve lies within the shaded region). F – females, M – males, A1 – monoantennary glycans, A2 – diantennary glycans, HM – high-mannose glycans, G0 – agalactosylated glycans, G1 – monogalactosylated glycans, G2 – digalactosylated glycans, F – glycans containing fucose, B – glycans containing bisecting *N*-acetylglucosamine, S0 – asialylated glycans, S1 – monosialylated glycans, S2 – disialylated glycans.

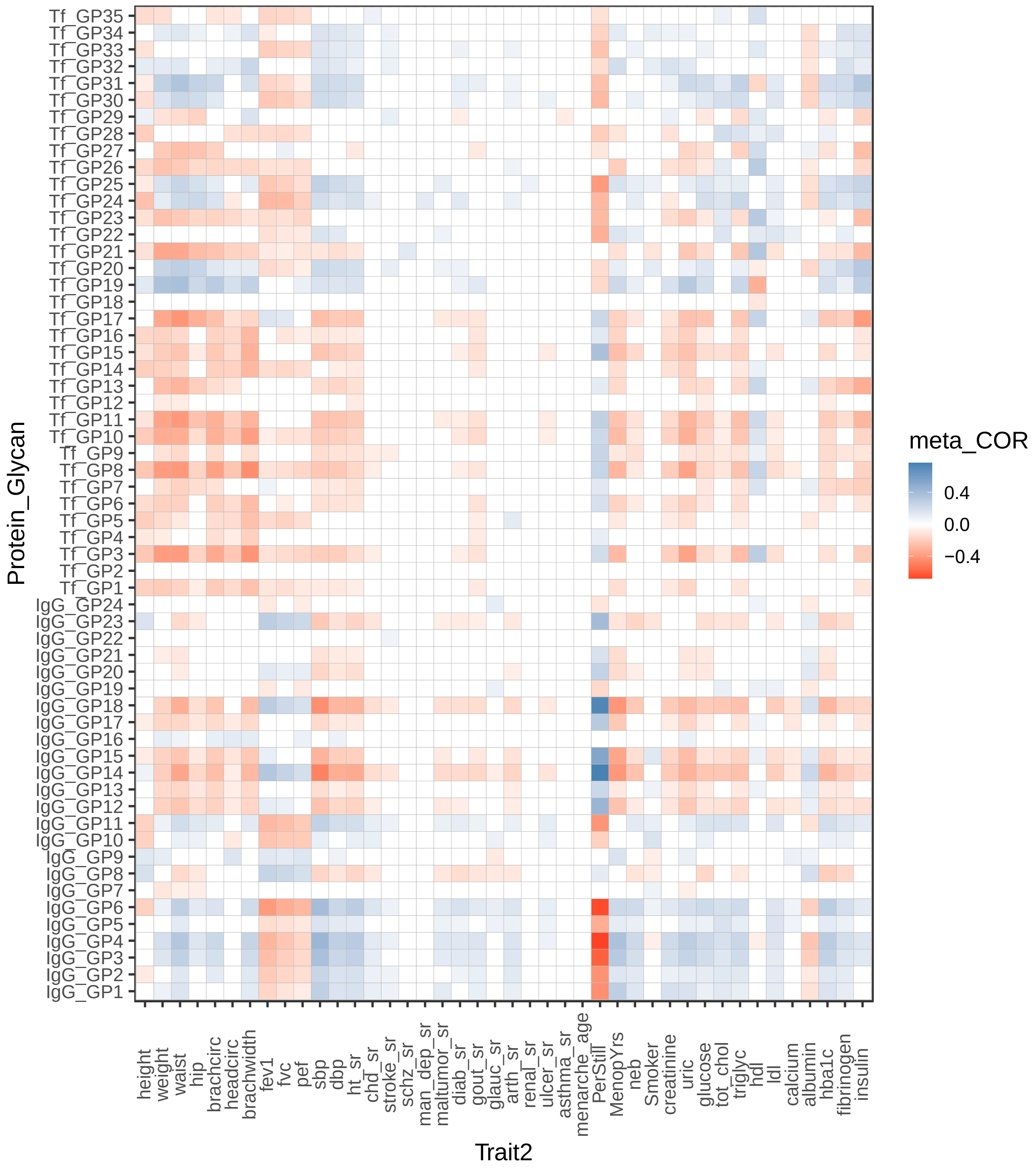

Supplementary Figure 9. Correlations between directly measured transferrin (Tf)/immunoglobulin G (IgG) N-glycan traits (GPs) and biochemical and physiological traits. Correlation analysis was performed on Korcula and VIKING cohorts separately and then combined using an inverse-variance weighted meta-analysis approach. Only statistically significant (adjusted meta p-value < 0.01, method = Benjamini- Hochberg) meta-correlations are shown, where blue color indicates a positive correlation, and red color indicates a negative correlation. Description of biochemical and physiological traits is given in **Supplementary Table 12**.

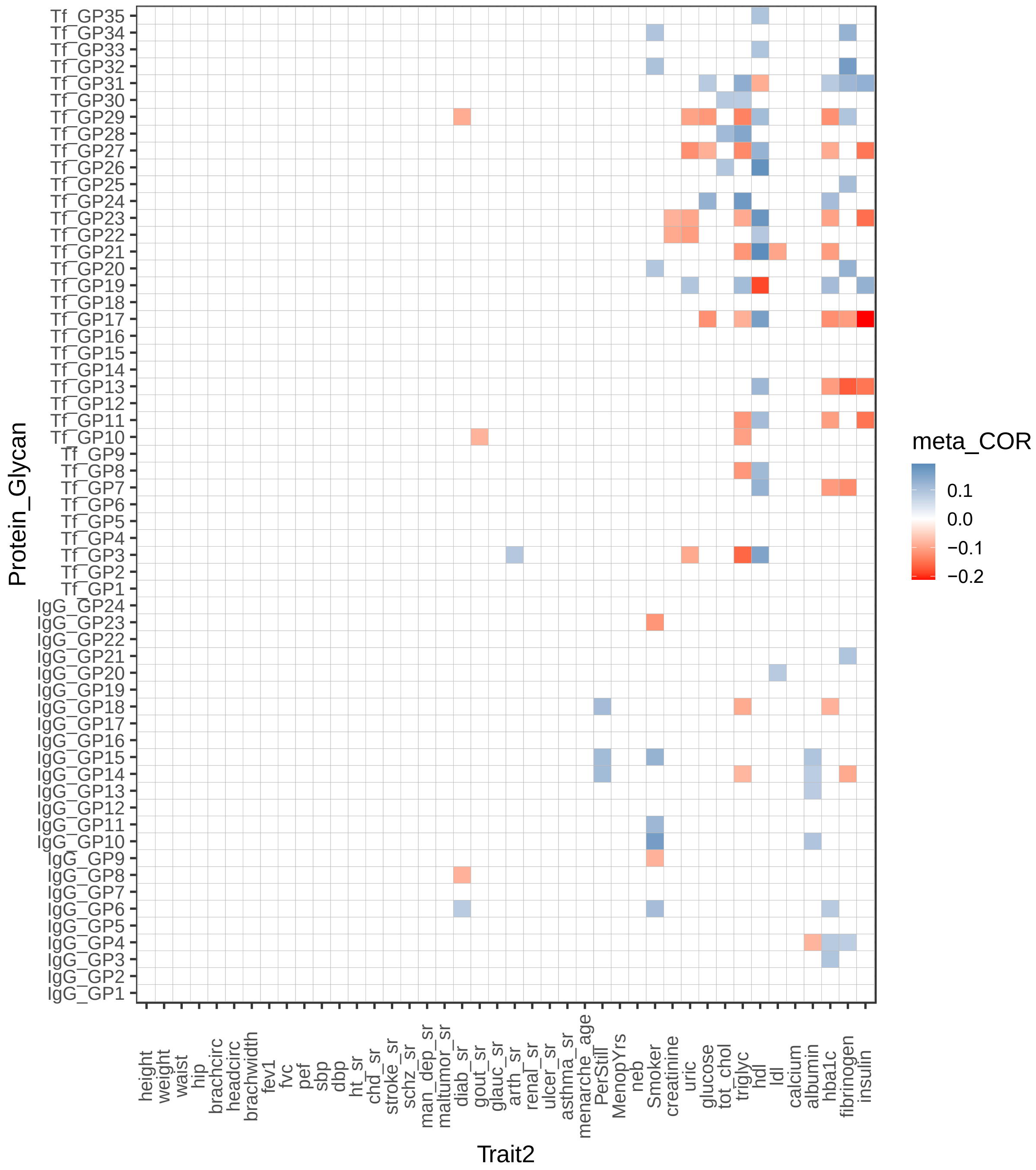

Supplementary Figure 10. Age-, sex-, and BMI-adjusted correlations between directly measured transferrin (Tf)/immunoglobulin G (IgG) traits (GPs) and biochemical and physiological traits. Correlation analysis was performed on Korcula and VIKING cohorts separately and then combined using an inverse-variance weighted meta-analysis approach. Prior to correlation analysis, glycan measurements were adjusted for age, sex, and BMI. Only statistically significant (adjusted meta p-value < 0.01, method = Benjamini- Hochberg) meta-correlations are shown, where blue color indicates a positive correlation, and red color indicates a negative correlation. Description of biochemical and physiological traits is given in **Supplementary Table 12**.

Supplementary Tables

Supplementary Tables 1-20 can be found in **Supplementary Data 1** file.

Supplementary References

1. Pučić, M. *et al.* High Throughput Isolation and Glycosylation Analysis of IgG-Variability and Heritability of the IgG Glycome in Three Isolated Human Populations. *Mol. Cell. Proteomics* **10**, M111.010090-M111.010090 (2011).
