# Supplementary material for: Comparative analysis of transferrin and IgG N-glycosylation in two human populations
Source: Commun Biol. 2023 Mar 23;6:312. doi: 10.1038/s42003-023-04685-6 (PMC10036557; doi:10.1038/s42003-023-04685-6)
Supplement: Supplementary file 3 — Description of Additional Supplementary Files [file 42003_2023_4685_MOESM3_ESM.docx]

**Description of Additional Supplementary Files**

**File name:** Supplementary Data 1
**Description:** Supplementary Tables 1-20 can be found in Supplementary Data 1 file.
