# Supplementary material for: Comparative analysis of transferrin and IgG N-glycosylation in two human populations
Source: Commun Biol. 2023 Mar 23;6:312. doi: 10.1038/s42003-023-04685-6 (PMC10036557; doi:10.1038/s42003-023-04685-6)
Supplement: Supplementary file 5 — Reporting Summary [file 42003_2023_4685_MOESM5_ESM.pdf]

nature portfolio

Corresponding author(s): Gordan Lauc

Last updated by author(s): Feb 28, 2023

Reporting Summary

Nature Portfolio wishes to improve the reproducibility of the work that we publish. This form provides structure for consistency and transparency in reporting. For further information on Nature Portfolio policies, see our Editorial Policies and the Editorial Policy Checklist.

Statistics

For all statistical analyses, confirm that the following items are present in the figure legend, table legend, main text, or Methods section.

n/a	Confirmed	
☐	☒	The exact sample size (*n*) for each experimental group/condition, given as a discrete number and unit of measurement
☐	☒	A statement on whether measurements were taken from distinct samples or whether the same sample was measured repeatedly
☐	☒	The statistical test(s) used AND whether they are one- or two-sided<br>*Only common tests should be described solely by name; describe more complex techniques in the Methods section.*
☐	☒	A description of all covariates tested
☐	☒	A description of any assumptions or corrections, such as tests of normality and adjustment for multiple comparisons
☐	☒	A full description of the statistical parameters including central tendency (e.g. means) or other basic estimates (e.g. regression coefficient) AND variation (e.g. standard deviation) or associated estimates of uncertainty (e.g. confidence intervals)
☐	☒	For null hypothesis testing, the test statistic (e.g. *F*, *t*, *r*) with confidence intervals, effect sizes, degrees of freedom and *P* value noted<br>*Give P values as exact values whenever suitable.*
☒	☐	For Bayesian analysis, information on the choice of priors and Markov chain Monte Carlo settings
☒	☐	For hierarchical and complex designs, identification of the appropriate level for tests and full reporting of outcomes
☐	☒	Estimates of effect sizes (e.g. Cohen's *d*, Pearson's *r*), indicating how they were calculated

Our web collection on statistics for biologists contains articles on many of the points above.

Software and code

Policy information about availability of computer code

Data collection	Glycomic data used in this study was generated using HILIC-UHPLC method. Acquity UHPLC instrument (Waters) was under the control of Empower 3 software, build 3471 (Waters). Glycans were also analyzed by MALDI-TOF-MS on an ultrafleXtreme MALDI-TOF-MS (Bruker Daltonics) under the control of Flexcontrol 3.3 software (Bruker Daltonics).
Data analysis	Empower 3 software was used to extract raw data from Acquity UHPLC instrument (Waters). Compass DataAnalysis 4.1, build 362.7 (Bruker Daltonics) was used for MALDI-TOF-MS spectra analysis. N-glycan structures were proposed using the GlycoStore database (www.glycostore.org) according to UHPLC-HILIC data, and GlycoMod Tool (https://web.expasy.org/glycomod/). GlycoWorkbench version 2.1, build 146 was used for MALDI-TOF-MS MS/MS spectra annotation. MaxQuant software 1.6.10.43 was used in proteomic analysis. Data were analyzed and visualized using the R programming language (version 4.0.2).

For manuscripts utilizing custom algorithms or software that are central to the research but not yet described in published literature, software must be made available to editors and reviewers. We strongly encourage code deposition in a community repository (e.g. GitHub). See the Nature Portfolio guidelines for submitting code & software for further information.

Data

Policy information about availability of data

All manuscripts must include a data availability statement. This statement should provide the following information, where applicable:

- Accession codes, unique identifiers, or web links for publicly available datasets
- A description of any restrictions on data availability
- For clinical datasets or third party data, please ensure that the statement adheres to our policy

There is neither Research Ethics Committee approval, nor consent from individual participants, to permit the open release of the individual-level research data underlying this study. The datasets analyzed during the current study are therefore not publicly available. Instead, the datasets generated and/or analyzed during the current study are available from the corresponding author on reasonable request and in line with the consent given by participants.

Human research participants

Policy information about studies involving human research participants and Sex and Gender in Research.

Reporting on sex and gender	In this study sex-based analyses were performed to determine to which extent Tf N-glycome was associated with sex. Additionally, in other analyses sex was included as covariate variable. Sex was determined based on self-reporting. Details on participants demographics is provided in Table 1.
Population characteristics	Korcula study was performed in the adult population of the island of Korčula, Croatia. All subjects were aged 18 and over. The Viking Health Study - Shetland (VIKING) is a family-based, cross-sectional study that seeks to identify genetic factors influencing cardiovascular and other disease risks in the population isolate of the Shetland Isles in northern Scotland. 2105 participants were recruited between 2013 and 2015, most having at least three grandparents from Shetland. Details on participants demographics is provided in Table 1.
Recruitment	In this study samples from two cohorts were analyzed - Korcula and VIKING cohorts. Korcula study was performed in the adult population of the island of Korčula, Croatia. The field work was performed in 2007 in the eastern parts of the island, focusing on the town of Korčula and villages Lumbarda, Žrnovo and Račišće. The sampling approach was convenient, with population-wide invites to non-institutionalized individuals living in the island. All subjects were aged 18 and over, and had signed informed consent before entering the study. The Viking Health Study - Shetland (VIKING) is a family-based, cross-sectional study that seeks to identify genetic factors influencing cardiovascular and other disease risks in the population isolate of the Shetland Isles in northern Scotland. Genetic diversity in this population is decreased compared to Mainland Scotland, consistent with the high levels of endogamy historically.2105 participants were recruited between 2013 and 2015, most having at least three grandparents from Shetland. All participants gave informed consent.
Ethics oversight	Ethical Committee of the Medical School, University of Zagreb and Multi-Centre Research Ethics Committee for Scotland; South East Scotland Research Ethics Committee, NHS Lothian; Ethical Committee of the Croatian National Institute of Transfusion Medicine

Note that full information on the approval of the study protocol must also be provided in the manuscript.

Field-specific reporting

Please select the one below that is the best fit for your research. If you are not sure, read the appropriate sections before making your selection.

☒ Life sciences ☐ Behavioural & social sciences ☐ Ecological, evolutionary & environmental sciences

For a reference copy of the document with all sections, see nature.com/documents/nr-reporting-summary-flat.pdf

Life sciences study design

All studies must disclose on these points even when the disclosure is negative.

Sample size	This is an observational study and samples were collected as samples of convenience. No statistical calculation of sample size was performed; sample size was determined based on availability.
Data exclusions	No data were excluded from the analysis.
Replication	Since the participants from the first cohort we analysed (KORCULA) were collected as the set of convenience, we wanted to add to the reliability of the conclusions and estimates by replicating the results in another independent cohort (VIKING).
Randomization	This is an observational study where experimental groups were not defined and where participants were not allocated into experimental groups. To control for the effect of experimental factors on glycan measurements, the blocked randomization of samples between experimental batches was performed. In such a design, every batch (block) maintains a constant distribution of known experimental and main biological factors.

Blinding

The persons performing sample preparation and analysis were unaware of the sample identity. This is an observational study where experimental groups were not defined and where participants were not allocated into experimental groups - all samples were analyzed with uniform set of techniques.

Reporting for specific materials, systems and methods

We require information from authors about some types of materials, experimental systems and methods used in many studies. Here, indicate whether each material, system or method listed is relevant to your study. If you are not sure if a list item applies to your research, read the appropriate section before selecting a response.

Materials & experimental systems

n/a	Involved in the study
☒	☐ Antibodies
☒	☐ Eukaryotic cell lines
☒	☐ Palaeontology and archaeology
☒	☐ Animals and other organisms
☒	☐ Clinical data
☒	☐ Dual use research of concern

Methods

n/a	Involved in the study
☒	☐ ChIP-seq
☒	☐ Flow cytometry
☒	☐ MRI-based neuroimaging
